# Diagnosis and treatment of type 1 diabetes at the dawn of the personalized medicine era

**DOI:** 10.1186/s12967-021-02778-6

**Published:** 2021-04-01

**Authors:** Ammira Al-Shabeeb Akil, Esraa Yassin, Aljazi Al-Maraghi, Elbay Aliyev, Khulod Al-Malki, Khalid A. Fakhro

**Affiliations:** 1 Department of Human Genetics-Precision Medicine Program, Sidra Medicine, P.O. Box 26999, Doha, Qatar; 2 Department of Genetic Medicine, Weill Cornell Medicine, P.O. Box 24144, Doha, Qatar; 3 College of Health and Life Sciences, Hamad Bin Khalifa University, P.O. Box 34110, Doha, Qatar

**Keywords:** Type 1 diabetes, Autoimmunity, Personalized medicine, Personalized treatment, Genomic Risk Score, Insulin therapy, Stem cells, Gene polymorphism, Stem cells, Gene therapy, Pancreatic β cells

## Abstract

Type 1 diabetes affects millions of people globally and requires careful management to avoid serious long-term complications, including heart and kidney disease, stroke, and loss of sight. The type 1 diabetes patient cohort is highly heterogeneous, with individuals presenting with disease at different stages and severities, arising from distinct etiologies, and overlaying varied genetic backgrounds. At present, the “one-size-fits-all” treatment for type 1 diabetes is exogenic insulin substitution therapy, but this approach fails to achieve optimal blood glucose control in many individuals. With advances in our understanding of early-stage diabetes development, diabetes stratification, and the role of genetics, type 1 diabetes is a promising candidate for a personalized medicine approach, which aims to apply “the right therapy at the right time, to the right patient”. In the case of type 1 diabetes, great efforts are now being focused on risk stratification for diabetes development to enable pre-clinical detection, and the application of treatments such as gene therapy, to prevent pancreatic destruction in a sub-set of patients. Alongside this, breakthroughs in stem cell therapies hold great promise for the regeneration of pancreatic tissues in some individuals. Here we review the recent initiatives in the field of personalized medicine for type 1 diabetes, including the latest discoveries in stem cell and gene therapy for the disease, and current obstacles that must be overcome before the dream of personalized medicine for all type 1 diabetes patients can be realized.

## Introduction

Type 1 Diabetes (T1D) is a potentially life-threatening multifactorial autoimmune disorder characterized by T-cell-mediated destruction of pancreatic β cells, resulting in a deficiency of insulin synthesis and secretion [1]. The incidence of T1D has been rising globally since the 1950s, with an average annual increase of 3–4% over the past three decades [2]. In particular, the incidence of childhood T1D is increasing, most rapidly in populations that previously had low incidence [3–5], and varying by ethnicity and race [4].

This worrying growth in T1D incidence has driven concerted research efforts to better understand the underlying risk factors, etiology, and pathology of the disease.

T1D has a largely heritable element, supported by a twin concordance rate of up to 70% [6] and of 8–10% sibling risk [7]. The bulk of risk is explained by difference at a several but strongly associated loci involving the HLA region “HLA class II, DQ and DR loci and HLA class I region” on chromosome 6p21 that account for ~ 50% of familial T1D [8, 9]. Genome‐wide association (GWAS) and candidate gene association studies have produced an abundance body of evidence and provided convincing support about other genes and loci external to the HLA region that protect or confer the risk for T1D [8, 10]. Single nucleotide polymorphisms (SNPs) comprising insulin gene (*INS*) presents ~ 10% of genetic predisposition of T1D [8, 11], cytotoxic T-lymphocyte–associated antigen (*CTLA*)-4 gene [12], protein tyrosine phosphatase non-receptor type 22 (*PTPN22)* [8, 13], nterferon induced with helicase C domain 1 (*IFIH1*) genes [14] and Interleukin-2 receptor alpha chain (*IL2RA*) [11]. This great genetic heritability generates the capacity for effective diagnostic discrimination if the most of genetic risk for T1D can be allocated [15, 16].

**Fig. 1:**
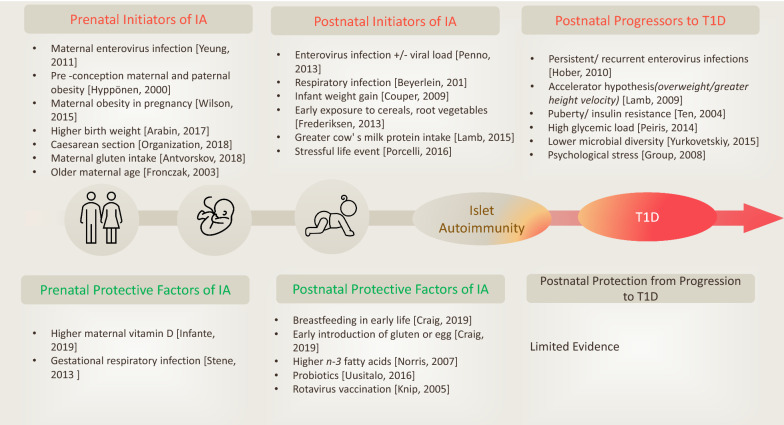
Environmental factors associated with initiation of, or protection from islet autoimmunity (IA) and progression to T1D. Adopted with permission from (Craig et al. 2019)

Prospective birth cohorts studies have facilitated the identification of potential triggers of islet autoimmunity (IA) and the natural history of progression to T1D [17–20]. Candidate triggers such as infections [21], early life diet [22], vitamin D levels [23], gut microbiota composition [24], vaccinations [25], pollutants and toxins [26], and geographic variation [27] when combine with genetic susceptibility [28] and specific epigenetic modifications [29–31], the perfect storm occurs and autoimmune destruction of pancreatic β cells is initiated (Fig. 1). These triggers required to be logged prospectively in well-designed studies instead of recollected retrospectively at the time of T1D diagnosis, couple of years later.

The plethora of factors that can lead to development and expression of T1D underpin the clinical heterogeneity of the disease. The gene polymorphisms and environmental triggers combinations that impact the risk of T1D and lead to the disease development are tremendously high [32]. Until now, this heterogeneity has not been taken into account and almost all T1D patients are treated with the standard approach of regular blood glucose monitoring combined with exogenous insulin replacement. However, the rising social and healthcare costs globally associated with T1D and its complications are providing the impetus for prioritizing more tailored approaches [33–35]. There is now increasing recognition of the opportunity to identify specific patient subgroups at different stages or with different driving factors of their early disease and prevent or even reverse their emerging T1D: this is the concept of personalized medicine. Personalized medicine is characterized by the mantra of "offering the right therapy at the right time for the right affected individual"; as an idea it is not new, but only recently has scientific and clinical research provided us with the necessary information and the means with which to apply it to novel treatment strategies for T1D.

In this review, we bring together the latest knowledge of the factors underpinning T1D heterogeneity in distinct patient groups and how these differences are being used to design personalized medicine approaches to diagnose, prevent, and hopefully treat the disease. We will discuss recent advances in gene therapy and stem cell-based treatments for specific groups of T1D patients, and will highlight key obstacles that must be overcome if further progress towards the goal of personalized medicine for all T1D patients is to be achieved.

## Personalized diagnosis of T1D

Although all patients with overt T1D exhibit pancreatic destruction and consequent dysregulation of blood glucose levels, not all cases of the disease are driven by the same factors or along the same timeline. Many patients experience a sometimes prolonged clinically silent phase in which it might have been possible to intervene and prevent or even reverse the course of disease. This knowledge has led to development of a staging classification system for T1D. Even once T1D is clinically evident, we are now beginning to appreciate that not all cases are the same, and that particular sub-types of the disease would benefit from distinct treatment strategies. We discuss both of these important advances within the field below.

### Staging classification system for T1D

By dissecting population- and individual-level risk factors for developing T1D, we now know that the disorder exists across developmental spectrum that can be categorized into distinct stages, and the likelihood of an individual developing clinically symptomatic status can be foreseen with considerable accuracy.

**Fig. 2:**
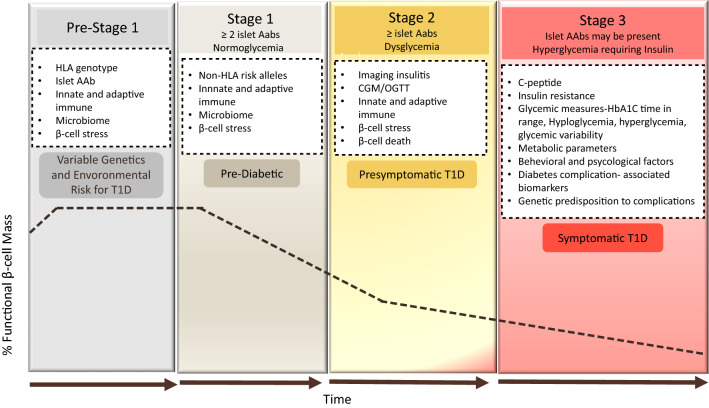
Development and staging of type 1 diabetes. T1D is characterized by a gradual loss of β-cell function (black dashed-dotted line) over time. As the disease progresses, beta cell function falls below the threshold required to maintain glucose control creating a requirement for insulin replacement therapy. Genetic and environmental risk are both included in the disease etiology. In stage 1, β-cell autoantibodies are persistent, but normoglycemia remains and there are no clinical symptoms. Throughout stage 2, the number of β-cell autoantibodies may induce dysglycemia but still without any diabetes symptoms. In stage 3, β-cell autoantibodies are predominant and clear symptoms of diabetes have emerged. In the white boxes are categories of biomarkers which could be leveraged to refine the staging paradigm, improve prognostic predictions, or subset individuals within a given stage of disease [38]. The specifics of these biomarkers are discussed in the text related to the relevant stage. The staging of T1D pathogenesis was proposed by Insel et al. [36] and the figure explanation was adapted from the same publication on addition to [36]© 2015 The American Diabetes Association

All cases are proposed to start with a period of "incubation" where exposure to defined and undefined driving factors creates the conditions for β-cell autoimmunity to emerge. When the process of ß-cell autoimmunity begins, the development towards clinical T1D can be classified into three distinct main stages: (I) asymptomatic ß-cell autoimmunity, defined by the presence of ≥ 2 types of autoantibodies such as GAD65 (GADA), zinc transporter 8 (ZnT8A), insulin (IAA), islet cell antibodies (ICA), insulinoma-associated proteins (IA-2A and IA-2β), with normoglycemia; (II) asymptomatic ß-cell autoimmunity, characterized by the presence of ≥ 2 types of autoantibodies but with dysglycemia, indicating functional damage to ß-cells; and (III) symptomatic T1D recognized by the symptoms of dysglycemia including polyuria or diabetic ketoacidosis (DKA) (Fig. 2). The sequence of events from emerging autoimmunity to dysglycemia and then to overt diabetes occurs along this predictable course, but the length of each stage may vary broadly between different individuals [36–38].

There are several valuable clinical outcomes for children monitored across prospective longitudinal natural history studies such as. Notably, those children have better metabolic markers at and soon after the clinical diagnosis stage, making the disease management relatively easier, reduce hypoglycemic incidents and delay the progress of the associated long-term complications. Rigorous diabetes management commenced afterward the diagnosis of symptomatic T1D increases the chance of a honeymoon phase [39], assists patients to preserve greater C-peptide ranges [40, 41], and reduce mortality rate [42], indicating that patients who are treated earlier will have improved long-term outcomes. In addition, genetically at risk children of DAISY (Diabetes Autoimmunity Study in the Young) cohort had lower HbA_1C_ levels maintained within the normal range, a figure much lower than the average HbA_1C_ levels of T1D children in the community [43, 44]. Also, only 3% of the DAISY children were hospitalized at T1D diagnosis compared to 44% of matched children in the community [44]. The DKA levels was detected in around 30% of the participants of the SEARCH for diabetes in youth study [45], while the same marker observed in lower prevalence in children screened positive for islet autoantibodies followed by German BABYDIAB and Munich family study [46].

Children followed by Diabetes Prediction in Skåne (DiPiS) study experienced decreased HbA_1C_ up to 24 months after the diagnosis against similar daily insulin dose requirements [47].

The predictable progression of T1D from early stages of autoimmunity to dysglycemia ahead of the symptomatic clinical disease could ease the design of reliable clinical trials using intermediate endpoint that require ~ 50% smaller sample size that those using T1D as the endpoint. In TrialNet natural history study, diabetes- related autoantibodies were analyzed in relatives of T1D patients in respect to elevated HbA_1C,_ decreased C-peptide following oral glucose tolerance test (OGTT) value as intermediate markers of T1D progression [48]. Also, the TrialNet CTLA4-Ig (abatacept) ongoing trial designed to test whether intervention with Abatacept could prevent or delay the development of abnormal glucose tolerance (AGT) in at-risk relatives of T1D patients [49]. Combined predictive risk score for an improved prediction of disease progression by incorporating fixed and variable factors (genetic, immunologic and metabolic markers) in newborn screening to prevent DKA and to enhance personalized risk predication for better T1D prevention trial selection [50, 51]. The crucial benefit of utilizing this staging system is to aid in development of innovative, stage-specific diagnostic and predictive biomarkers, support the design of clinical trials that utilizing the available data on risk profiles and individuals’ pre-symptomatic classification to design therapies specifically targeted to each phase of disease and ultimately, practice of personalized medicine approaches to avert symptomatic T1D. Future research will be needed to identify the main drivers of the transitions between stages in order to identify novel therapeutic targets to prevent the emergence of T1D in high-risk populations.

### Diagnostic sub-groups within symptomatic T1D

Diagnosis of T1D has historically been made on the basis of detecting blood glucose dysregulation; however, this has led to patients with diverse underlying pathologies being grouped, and treated, together. Evidence of β-cell destruction via the presence of anti-islet-autoantibodies (which may recognize insulin, Glutamic Acid Decarboxylase 65(GAD65), zinc transporter isoform 8 (ZnT8), or islet cell antigen (ICA512) and the age at which initial autoantibodies were detected are important factors that characterize the “classical” etiological subtype of T1D. However, less frequently, hypoglycemia might be caused by loss of function or de novo mutation in a sporadic gene, giving rise to monogenic diabetes, which represents 3% of all diabetes cases in children and adults [52]. The heterozygous activation of genes encoding the ATP-sensitive potassium-channel subunit Kir6.2 reported to cause permanent neonatal diabetes in addition to some neurological abnormalities in some affected individuals. Distinguishing monogenic diabetes from T1D is crucial for accurate diagnosis, applying the correct treatment “such as sulfonylureas in Kir6.2 mutation”, and in the future, stratifying these patients into a group most likely to benefit from gene therapy targeting the mutation.

The aim of increasing correct diagnosis of classical versus monogenic T1D has been assisted by the introduction of the genomic risk score (GRS), which assesses an individual’s risk of T1D based on their possession of a collection of multiple (10–40) T1D risk variants [53, 54]. The GRS also effectively identifies those individuals with early-onset or pre-clinical T1D who show more autoimmunity and fewer syndromic features in comparison with those of monogenic diabetes [55]. The sensitivity and specificity of the T1D-GRS exceeds 80% [55], but this figure might reasonably expect to be increased when the GRS is combined with the available clinical data and autoantibody results. Accordingly, incorporating the T1D-GRS into strategies aimed at intervening in the pre-symptomatic T1D stages noted above (Fig. 1, [31, 56–79]) is likely to prove productive in the development of personalized diabetes-preventative therapies targeting either mutational correction or prevention of overt autoimmunity.

Somewhat surprisingly, T1D and type 2 diabetes (T2D) are often distinguished based on whether the person exhibiting blood glucose dysregulation is young and a healthy weight (T1D-typical), or instead an older adult with obesity (T2D-typical). However, these two manifestations have different causes and medication requirements [80]. Research in 2017 found that approximately 40% of people who developed T1D after the age of 30 were initially diagnosed and treated for T2D [81]. Given the potentially life-threatening nature of insulin-deficiency status [81, 82], these findings call for increased use of autoantibody testing to discriminate T1D and T2D, and widespread recognition of the fact that clinical features alone cannot reliably distinguish these two conditions.

Current advances in affordable high-throughput genomic and molecular deep phenotyping technologies have pushed the rise of “next-generation epidemiology” with a more systematic focus than before. In particular, deep phenotyping can be described as the precise and broad analysis of phenotypic data to aid in identifying disease biomarkers that assist the prediction, prevention and disease monitoring [83]. Recently, an integrative multi-omics approaches were used on the Environmental Determinants of Diabetes in the Young (TEDDY) children, a prospective longitudinal birth cohort created to study T1D by following children with high genetic risk [84]. The analysis identified a multi-omics signature that able to predict the IA before seroconversion in one year, in addition, defects in lipid metabolism, problems with nutrient absorption, reactive oxygen species (ROS) detected prior to the IA progression.

In conclusion, identification of high risk for T1D genetic groups in the pre-symptomatic stages, coupled with the use of autoantibody testing, GRS and molecular deep phenotyping through utilizing the advanced integrative data analysis, could support the development of approaches for early diagnosis and treatment of T1D in both symptomatic and pre-symptomatic patients. This strategy could form the mainstay of accurate “personalized diagnoses” moving forward. Understanding the genetic etiology and specific pathophysiology of these distinct patient groups within the T1D family will be necessary for the rationale design and application of personalized therapies in the future.

## Personalized treatment of T1D

Progress in recognition of the need for personalized diagnosis in T1D has been accompanied by intense research efforts towards personalized therapies. Before the discovery of insulin in 1921, it was remarkable for T1D patients to live more than one or two years after disease onset: one of the twentieth century’s utmost medical breakthroughs, insulin replacement, is still the mainstay of treatment for the vast majority of T1D patients today. That said, innovative ways of achieving improved insulin-mediated glycemic control are becoming accessible to patients, while tissue transplants, genetic modification and stem-cell therapies are showing promise in pre-clinical models and human trials in specific sub-groups of patients. In this section we will discuss the “old and new” of T1D therapies and moves towards personalization to increase treatment efficacy.

### Insulin and combination drug therapies

By far, the most common T1D treatment approach is manual testing of blood sugar levels followed by sub-cutaneous injections of insulin, repeated throughout the day. Insulin pumps may be used in place of traditional injections [85]; these have the advantage of being able to continuously infuse small amounts of insulin sub-cutaneously, helping those patients with difficult-to-control glucose levels to better treat their disease. This is especially the case when coupled with continuous glucose monitoring (CGM) technology, which has been shown to improve control of blood glucose, thereby reducing long-term risks of diabetic complications [86, 87].

Taking the combination of CGM and continuous insulin infusion to the next level is the advent of the artificial pancreas. By utilizing a CGM coupled via a control algorithm to an implanted insulin pump, people with T1D can achieve improved glycemic outcomes while reducing the burden of self-management [88–90]. A closed-loop artificial pancreas approach removes the need for the patients to manage their dosages at all, and some models also incorporate the pancreatic hormone glucagon, enabling glucose-responsive hormone delivery guided by real-time glucose sensor readings. This approach has the potential to accommodate highly variable day-to-day insulin/glucagon requirements. There will be a shift toward systems that offer more personalization, and individualization of adjusting parameters, glucose set algorithm aggressiveness proposed to be individualized including the daily targets [91] that can ensure tight glycemic control in affected patients [92, 93]. Despite these advantages, still relatively few T1D patients are using an artificial pancreas, with the main obstacles being cost of the equipment, the need for a training infrastructure for users and clinicians, and a lack of clarity around which patient groups would benefit most from this technology (reviewed in [92]). In this case the technology has preceded the clinical sub-group analysis required to identify the patient groups who are most suited to the approach, calling for urgent research in order to fully exploit this important advance in insulin-replacement therapy.

Alongside developments in insulin replacement therapy, there has been a focus on identifying other drugs that can be combined with insulin to reduce hyper/hypoglycemia and improve metabolic variables without increasing adverse events (reviewed in [94]). Obese/T1D patients who predisposed to hypoglycemia and others with residual β-cell function could benefit from non-insulin antidiabetic drugs for future clinical trials [94, 95]. Of these, promising candidates include metformin [96] and pramlintide, which have a role in glycemic control in both T1D and T2D and can modestly reduce triglyceride levels in T1D patients, as well as lowering hemoglobin A1c (HbA1_c_) and supporting weight loss [97]. In addition, glucagon-like peptide-1 receptor agonists (GLP-RAs) combined with insulin can reduce the daily bolus insulin dose required and improve glucose control and weight loss [98]. The incretins glucagon-like peptide 1 (GLP-1) is gut-derived hormone secreted upon food ingestion. The key physiological actions of GLP-1 are to accelerate nutrient-induced insulin release and inhibit glucagon secretion, in that way contributing to regulate postprandial glucose excursions [99]. In addition, other functions represented by inhibition of gastrointestinal motility and therefore works as “enterogastrone”, a hormone released by the lower gastrointestinal tract in reaction to lipids intake that constrains the caudal motion of the guts of chyme [100]. GLP-RAs used peripherally or centrally reduce food intake and escalate glucose-stimulated insulin secretion. The enzyme dipeptidyl peptidase-4 inhibitors (DPP-4) prevents the inactivation of GLP-1 and an adjunct therapy in a closed loop-system that can reduce postprandial blood glucose levels [101] and can significantly reduce the daily insulin dose but not the HbA1c level or the risk of hypoglycemia [102]. The DPP-4 enzyme is widely released in multiple organs and acts by cleavage of the two NH_2_-terminal amino acids of bioactive peptides if the second amino acid is alanine or proline [103]. It functions through affixed transmembrane fragment and a soluble protein. Both transmembrane fragment and soluble DPP-4 apply catalytic cleavage which alternatively inactivates peptides or generates new bioactive moieties that may exert competing or unique functions. Finally, sodium-glucose co-transporter inhibitors (SGLTi) are associated with improved glycemic control and a reduced insulin dosage leading to lower rate of hypoglycemic episodes [104]. In non-diabetics, approximately, 180 g of glucose is filtered diurnal through the renal glomeruli and is then re reabsorbed in the proximal convoluted tubule (PCT). This mechanism attained by inactive transporters, specifically, facilitated glucose transporters (GLUTs), and by active co-transporters, precisely, sodium-glucose co-transporters (SGLTs). SGLT1 and SGLT2 are considered most important out of the six identified SGLTs [105]. SGLTi acts by inhibiting SGLT2 in the PCT to block glucose reabsorption and ease its secretion in urine. The plasma glucose levels drop resulting in an improvement in the entire glycemic parameters [106].

In summary, traditional and combined approaches to insulin therapy remain important tools in the treatment of T1D, but they do not represent a cure and may not be able to achieve the level of glucose control necessary to avoid long-term complications arising from diabetes. Automated full closed-loop systems that can be programed to automatically manage meals may substantially benefit from faster acting insulins with a shorter duration of action. Proposing automatic flexibility to the individual’s changes not only daily patterns of insulin sensitivity but also to mechanically adjust to changes developing from illness, workout practices, eating routines and menstrual cycles. With the applications of machine learning (artificial intelligence), (AI), the future devices with the AI technologies could achieve the above relationship and to provide treatment suggestions and decisions based on the available data input. A unique and individualized predictive and decision support models using complex machine learning software and algorithms developed for insulin pumps for easier use and much more spontaneous daily life. Recently, Tyler et al. (reviewed in [107]) reported an algorithm for early recognition of unsafe insulin regimens which could be useful for improvement the glycemic results and minimize the dangerous complications of T1D [107]. Briefly, the algorithm offers weekly insulin dosage recommendations for adult patients with T1D using multiple daily injections protocol of long-acting basal and short-acting bolus insulin [108]. The hyperglycemia or hypoglycemia causes identification performed through validated single and dual hormone mathematical models that demonstrate a virtual platform of T1D patients [109]. The novel “virtual platform” employed to generate glucose observations used to train “decision making system”, which appeared to be in agreement with the endocrinologists’ decision of 67.9% when confirmed on actual human data [107, 110]. In conclusion, such data provides guidance to physicians and T1D patients in effective use of insulin pumps data including but not limited to insulin dosing adjustments and other treatment decisions. It’s worth to mention how crucial that both physicians and diabetic patients understand the usefulness and limitations of insulin pumps and related treatment technologies. Sustaining the relationship between both will remain a critical factor in safe, thriving T1D treatment technology use.

### Gene therapy

**Fig. 3:**
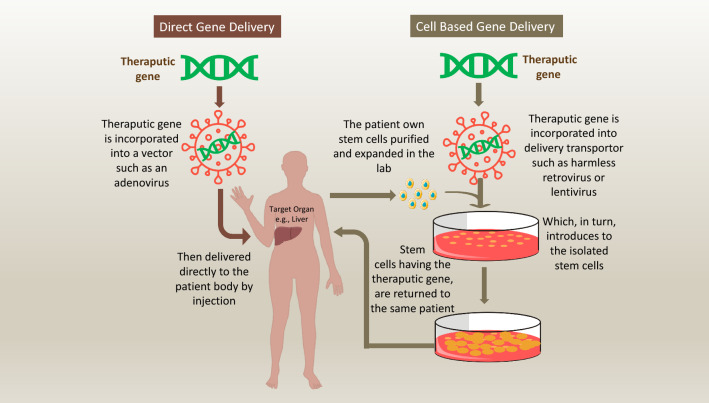
How genes are delivered to the human body during gene therapy approaches. Gene therapy have utilized two major approaches for transferring therapeutic transgenes into recipients 'body. First approach, is by direct infusion of the therapeutic gene into human body through a vehicle. Altered viruses often used for delivering the gene into specific human cell types. This method is inexact as it is limited to specific cell types that the viral vehicle can infect. Nonviral vehicles for directly delivering genes into cells are also being explored, including the use of plain DNA and DNA wrapped in a coat of fatty molecules known as liposomes. Th second approach utilize a living cells to transfer the therapeutic transgenes into recipients 'body. The transferring cells often a type of stem cell that removed from the body, and the therapeutic transgene is presented to them through direct transfer method. The genetically altered cells then grow and multiply before infused back to the recipient

Given the strong genetic component of T1D development, gene therapy offers a promising alternative to insulin injection for T1D treatment. Gene therapy is the procedure of transporting or manipulating genetic substances inside the cell as a therapeutic technique to cure disease [111]; it aims to modify faulty genes that are accountable for disease progression and thereby prevent disease onset or reverse its development (Fig. 3). The three key methodologies in gene therapy are: (I) introducing a new gene into the body (II) substituting defective genes with functional genes, and (III) deactivating the faulty genes triggering the disease [112]. Pre-clinical trials of gene therapy have now been tested with the aims of preventing or delaying onset of T1D, correcting insulin deficiency, promoting β-cell proliferation and survival, modulating the immune/inflammatory response or inducing insulin secretion by non-β cells (reviewed in [113]).

Over the last few decades, gene transfer trials for the treatment of inherited or acquired diseases have mainly been performed in mice models. Non-obese diabetic (NOD) mouse has been the main animal model for studying autoimmune T1D. A key element of NOD model is the presence of spontaneous autoimmunity and T1D. The incidence of T1D is higher in females in NOD mice, [114, 115], and is stated to have a minor prevalence in males in humans [116, 117]. Like human, NOD mice develop autoantibodies and show elevated levels of autoreactive T-Cells ahead of disease onset [118–120]. The targeted antigens of β cell are also similar of both species, however, in the NOD mouse, the insulin seems to be the initiating antigen, while in human T1D, several antigens thought to be involved in this stage [118]. Gradual β cell death or malfunction, and autoimmune phenotypes shadowed by the onset of hyperglycemia exist in both human and NOD mouse [121], however, the appearance of pathogenic T cells have been noticed at 5-week-old NOD mice followed by insulitis throughout the pancreas by 12 weeks, reflecting the very aggressive nature of disease onset hits in shortened timeline (weeks only), compared to slower onset in humans (years after the autoantibodies appearance) [122, 123].

The paradoxical assumption is that preventing T1D in NOD mice does not certainly convey what triggered the disease nor how to converse it. The NOD mouse model could be suitable to understand the genetic and immunologic features and causes of T1D including reversing the hyperglycemia when occurs. The model could serve as an approach to identify causative gene variants that can be tailored to discover novel therapeutic approaches for reversing new-onset T1D.

One particularly interesting strategy is the induced over-expression of insulin-like growth factor 1 (IGF1), which regulates immune functions and enhances the survival and proliferation of β-cells. Non-obese diabetic (NOD) mice spontaneously develop diabetes from around 10 weeks-of-age; however, when 4-week-old NOD mice underwent intra-ductal injection of an adeno-associated virus (AAV) encoding IGF1 to specifically transduce pancreatic cells, normoglycemia remained in 80% of these mice at week 28 [124]. Importantly, the same study also showed that treating NOD mice with the IGF1-encoding virus at 11 weeks-of-age, by which time significant β-cell destruction was evident, was able to re-establish lasting normoglycemia in 75% of mice [124].

In other animal studies, induced expression of regenerating islet-derived protein 3 gamma (Reg3g) has been reported to be able to regenerate β cells and preserve the cells despite autoimmune attacks [125, 126]. Alongside, another study demonstrated the dynamic regulation of blood glucose levels in a model of T1D by stimulating the expression of glucose 6-phosphatase (G6Pase) in the liver of diabetic rats [127]. Here, expression of the G6Pase gene was induced by rising glucose levels and inhibited by insulin expression; in addition to achieving normoglycemia within a few hours of eating, no hypoglycemia was observed in the tested animals [127].

Gene therapy can also be used to induce insulin production in non-β-cells. Initial studies conducted on genetically engineered intestinal K cells [128] and hepatocytes showed that these cells were sensitive to glucose and could be induced to produce insulin. More recently, Jaen et al. demonstrated that a single injection of an AAV encoding insulin and glucokinase genes into skeletal muscle of diabetic dogs was able to induce metabolic normalization and normoglycemia lasting 8 years [129]. This study represents an important safety and efficacy step forwards for diabetes gene therapy, as although AAV vectors have been trialed in humans, their therapeutic use for gene transduction has yet to be tested clinically. There are concerns that transduced cells might be susceptible to recurring autoimmune attack, so enduring autoimmune protection must be demonstrated [130, 131]. It is also possible that the viral vectors themselves might trigger an immune response that could worsen the disease condition [132], though Jaen et al. did not report any evidence of this in their study [129]. Modifications to the AAV vectors might hold some of the answers: in response to concerns that constitutive over-expression of insulin might risk hypo-glycaemia, one group has developed a Tet-off regulatable AAV vector for insulin expression that was able to both induce the expression of human insulin in diabetic mice, and be reversibly switched off to reduce insulin levels [133]. Thus, fine tuning of viral vectors combined with more long-term studies will be required to move towards vector-mediated reinstatement of insulin production in human patients.

In addition to induced insulin expression, several studies have looked at other targets implicated in T1D pathogenesis. For example, Klotho is an anti-aging gene that is expressed in pancreatic islets in mice [134] and humans [135]; a Klotho deficiency is linked with β-cell apoptosis, and reinstating its expression in mice under the control of a β-cell-specific promoter led to protection of β-cell function [134]. In human islet cells, treatment with the T1D drug gamma-aminobutyric acid in vitro significantly increased Klotho expression [136], indicating the possible clinical potential for this approach. A study by Flotyńska et al. demonstrated the relationship between fibroblast growth factor 23 (FGF23)/ Klotho system as a player in the human body metabolism, in addition to promoting longevity [137]. Despite the improvements in diabetes treatment, the long-term complications remain a big problem. The interesting correlation between the FGF23/Klotho system concentration and T1D management, duration, insulin resistance, and complications development require further attention and could be a predictor of cardiovascular risk in diabetic patients [138]. Combining gene therapy with immune modulation may also be promising. When NOD mice were pre-treated with anti-T-cell receptor β chain monoclonal antibody followed by hepatic gene therapy with Neurogenin-3 (which determines islet lineage) and the islet growth factor betacellulin, the researchers observed sustained induction of insulin-producing cells in the liver that achieved enduring reversal of new-onset or overt diabetes [139].

The discovery of β-cell mitogenic effects of *ANGPTL8* (Angiopoietin Like 8), which was renamed “Betatrophin” to underline its effect on β cell replication, initially, created large interest but consequently, have been subjected to substantial debate regarding its anticipated mitogenic effects [140]. The initial findings proposed that the over expression of *ANGPTL8* in mice model stimulated a 17-fold increase in pancreatic β-cell proliferation [140, 141]. Consequent research studies in mice disputed this statement as no substantial evidence could be observed to support the direct effects of ANGPTL8 on beta-cell proliferation [140, 142, 143], Therefore, *ANGPTL8* is not considered as a potential agent for diabetes intervention although some reports supported the initial observations in rats [144]. In a study performed by Chen et al. (reviewed by [144]), targeted gene delivery approach has been used to deliver human *ANGPTL8* gene plasmids to different organs of normal adult rats including the pancreas, liver and skeletal muscles and compared the efficiency of beta β cell replication induced by *ANGPTL8* gene using the rat model of streptozotocin (STZ)-induced diabetes. The improvement in glucose tolerance plus the elevated fasting plasma insulin levels were directly associated with β cell proliferation. A novel gene therapy technique used here through targeting the transfer of non-viral DNA to the pancreatic islet by using ultrasound-targeted microbubble destruction (UTMD) beside an altered insulin promoter [140, 145]. UTMD considered as promising method for target-specific gene delivery, and it has been successfully investigated for the treatment of many diseases in the past decade including cardiovascular disorders and cancer.

A novel approach to gene therapy for T1D involves targeting post-transcriptional modifications that give rise to pathogenic splice variants. Cytotoxic T-lymphocyte–associated antigen-4 (CTLA-4) is an immune-modulatory protein where expression of different forms has been linked to T1D susceptibility or resistance in T1D patients [146] and some other autoimmune diseases [147]. To modulate the immune response leading to T1D onset, Mourich et al. employed an antisense-targeted splice-switching approach to produce CTLA-4 splice forms in NOD mouse T-cells [148]. In this study, when the antisense approach was used to mask pre-mRNA splice recognition sites and redirect the splicing machinery to skip selected exons, induced over-expression of the protective ligand-independent form of CTLA-4 protected NOD mice from disease [148].

Lastly, while these studies clearly indicate the exciting potential of in vivo gene therapy, the process remains complex, in addition, the possible toxicity of the viral vectors and the improvements needed to the delivery systems to achieve the maximum levels of gene expression still under development [125]. That said, twenty gene and cell-based gene therapy products have now been licensed for the treatment of human cancers and monogenic disorders “e.g., Neovasculgen (Vascular endothelial growth factor, VEGF), Glybera (lipoprotein lipase, LPL^S447X^ gene), Defitelio (single-stranded oligonucleotides-VOD), Rexin-G (Retroviral vector encoding cyclin G1 inhibitor), Onpattro (RNAi-transthyretin gene)” and clinical trials in these diseases are ongoing [149]. There is real hope that effective approaches to direct gene therapy for T1D patients, particularly those with monogenic T1D, will be developed in the near future, building on its success in other conditions.

### Stem cell therapies

Perhaps the most promising innovation in T1D therapy has been the exploration of the potential of stem cells. This unique population is able to self-renew indefinitely, form single cell-derived clonal cell populations, and differentiate into various cell types [150]. Stem cells from diverse sources have now been investigated for their potential in β-cell regeneration, as discussed below.

#### Embryonic stem cells

Embryonic Stem Cells (ESCs) are derived from the undifferentiated inner cell mass of human embryos and have the advantage of being completely pluripotent. Several different approaches to generating insulin-producing cells (IPCs) from ESCs have been explored. Human Embryonic Stem Cells ESCs (hESCs) in feeder-free cultures avoid the risk of animal pathogen transfer and are readily scalable, making this approach best-suited to clinical use [151].

Kroon et al. instructed the differentiation of hESCs by directly overexpressing essential β-cell transcription factors (TFs) including Pancreatic and Duodenal Homeobox 1 (PDX1), SRY-Box Transcription Factor 9 (SOX9), Homeobox protein Nkx-6.1 (NKX6.1) and Neurogenin 3 (NGN3; following engraftment into diabetic mice, the resulting cells recapitulated key features of pancreatic β-cells and protected against hyperglycemia [152]. Subsequently, an important step forwards in the use of hESCs for T1D therapy occurred when scientists from the University of British Columbia developed a seven-stage protocol that efficiently converted hESCs into IPCs. This protocol generated endocrine cells with insulin content similar to that of human islet cells and that were capable of glucose-stimulated insulin secretion in vitro as well as rapid reversal of diabetes in vivo in mice [153]. Additional studies have highlighted the possible roles of other growth and extracellular matrix factors, including laminin, nicotinamide, insulin [154], and retinoic acid [155] in the generation of IPCs from ESCs, but these findings have yet to be integrated into a combined approach suitable for clinical use.

hESCs also have the potential to generate cells uniquely tailored for the recipient. Recently, Sui et al. showed that transferring the nucleus of skin fibroblasts from T1D patients into hESCs gave rise to differentiated β-cells with comparable performance to naturally occurring β-cells when transplanted into mice [156].

Despite the promise of hESCs, great concern around their potential to initiate teratomas has largely limited their clinical exploration in T1D. However, Qadir et al. recently demonstrated a means of overcoming this risk: the authors modified hESCs to include two suicide gene cassettes, whose expression results in cell death in the presence of specific pro-drugs [157]. Their method is designed to provide a double fail-safe control, such that I) only IPCs survive selection; and II) cells that may de-differentiate after transplantation can be eliminated. Furthermore, ensuring that undifferentiated cells are sensitive to two pro-drugs makes it less likely than any tumorigenic cells would survive or became resistant [158].

#### Human pluripotent stem cells

Naturally, Human Pluripotent Stem Cells (hPSCs) are immature cells that have the capacity to become nearly any cell type in the body. Accordingly, there has been much research interest in using them to regenerate a wide range of tissues, including the pancreas. Under the control of specific growth factors, signaling pathways and activating/inhibitory molecules [159, 160] the steps of pancreatic cell differentiation have been successfully recreated in vitro.

The importance of this approach is its potential to generate a ready supply of in vitro-differentiated β-cells for transplantation into T1D patients. Recent studies have reported the successful differentiation of β-like cells with enhanced function from pancreatic progenitors through modulating Epidermal growth factor beta (EGF-β) signaling and cellular cluster size, giving rise to stem cell-derived β-cells with the ability to express key β-cell markers and insulin [161, 162]. What remains unclear is how well these in vitro-derived cells will function in vivo*,* but this is nonetheless a promising first step.

#### Hematopoietic stem cells

Taking a different approach, myeloablation coupled with autologous Hematopoietic Stem Cells (HSCs) transplantation aims to halt the autoimmune destruction of the pancreas and reestablish tolerance. The first autologous HSCs transplantation in a T1D patient was executed by the Voltarelli’ group in 2007: 15 patients aged between 14 add 31 years, and with recent T1D onset (previous 6 weeks) diagnosed by clinical findings, hyperglycemia and GAD65 autoantibodies were involved in the study [163]. When these patients were treated with autologous HSCs, most achieved insulin independence with good glycemic control lasting until the final 29.8-month follow-up, together with a notable increase in β-cell function [164]. Autologous HSC transplantation has also been used successfully to treat diabetic sequelae, including vascular complications [165] and retinopathy [166]. Other studies have focused on understanding the mechanisms underlying successful HSCs transplantation in T1D: for example, Ye et al., found that autologous HSC treatment was associated with the inhibition of T-cell proliferation and pro-inflammatory cytokine production [167]; while Xiang et al. uncovered a critical role for the remaining functional β-cells on the autologous transplant of HSCs [168].

Despite the evident successes of autologous HSCs transplantation for T1D, various complications can occur, ranging from relatively mild symptoms such as febrile neutropenia, nausea, and alopecia to more severe complications such as de novo autoimmunity and systemic infections, which in one case resulted in death [169, 170]. The development of new strategies involving autologous HSCs therapy for newly-diagnosed T1D patients coupled with appropriate and effective use of immunosuppressive drugs will be crucial to maximize the frequency and function of T and B regulatory cells, while minimizing the activity of autoreactive islet-specific T and B memory cells. In this way, we should be able to improve treatment outcomes in T1D patients undergoing transplantation.

#### Mesenchymal stem cells

Mesenchymal Stem Cells (MSCs) are multi-potent stromal cells able to differentiate in vitro into a range of cell types; characteristically adipocytes, chondrocytes, myocytes, and osteoblasts [171]. MSCs are relatively easy to isolate from different sources in the body and numerous studies have assessed their use in T1D therapy.

Historically, the bone marrow has been the main source of MSCs [172]. Xie et al. first trialed generating IPCs from T1D patients’ bone marrow MSCs (BM-MSCs) and showed the co-expression of insulin and C-peptide in cells injected into diabetic mice, leading to attenuated hyperglycemia [173]. Alongside, genetically-modified human BM-MSCs expressing VEGF and PDX1 reversed hyperglycemia in more than half of diabetic mice and enabled survival and weight maintenance in all animals [174]. These promising pre-clinical results led to human trials: when BM-MSCs were injected into the splenic artery of T1D patients, they induced an increase in C-peptide levels that was maintained for 3 years; unfortunately, this had no significant effects on glycemic control due to insufficient production of insulin by the grafted cells [175]. Since then, new methods have been developed aiming to improve in vivo outcomes. For example, Zhang et al. co-cultured BM-MSCs with pancreatic stem cells which led the MSCs to adopt a pancreatic islet morphology; when these cells were injected into diabetic rats they attenuated glycated albumin levels and significantly increased serum insulin and C-peptide [176].

The main disadvantage of BM-MSCs is the difficulty in isolating the cells and the morbidity associated with the procedure. These issues led to interest in the use of Muscle-Derived Stem/Progenitor Cells (MDSPCs), which exist in skeletal muscle and have the capacity for long-term proliferation, are resistant to oxidative and inflammatory stress, and show multi-lineage differentiation potential [177]. To investigate the therapeutic potential of autologous MDSPCs transplantation for T1D, Lan et al. applied a four-stage MDSPCs differentiation protocol to generate IPCs in vitro and injected them into diabetic mice: these β-cell-like-cells effectively improved hyperglycemia and glucose intolerance and increased the survival rate in diabetic mice without the use of immunosuppressants [178].

Building on the promise of BM-MSCs and MDSPCs, researchers sought an equally potent but more abundant and easily accessed source of stem cells. Adipose-Derived Stem Cells (ADSCs) have recently been explored for T1D treatment, and have the advantage over MDSPCs of being readily accessible and harvested, even in older patients [179]. IPCs differentiated from ADSCs show significant expression of β-cell markers, insulin and c-peptide following transfer into diabetic mice [180]. In 2019, IPCs derived from ADSCs using a novel three-dimensional (3D) xenoantigen-free protocol were shown to exhibit key features of pancreatic β cells in vitro and differentiated into IPCs in diabetic nude mice in vivo [181]. Another study showed the potential for combining ADSCs treatment with gene therapy by transducing ADSCs with a furin-cleavable insulin gene (INS-FUR), which led to enhanced insulin expression in the differentiated adipocytes, and alleviated hyperglycemia in diabetic mice [182].

Removing the need for adult stem cell donors completely, the umbilical cord is now used as a successful alternative stem cell source for regenerative medicine. Umbilical cord blood (UCB) is rich in HSCs, can be easily harvested without the need for interventions, and also contains a large number of naive functioning T-regulatory cells (Treg) with the potential to reduce autoimmunity [183, 184]. Moreover, the MSCs within UCB (UCB-MSCs) have high proliferative capacity, are easily bankable and have low tumorigenicity [185]. Together these features are making UCB-MSCs the preferred option for potential T1D cell-based therapies. Studies in animal models have showed encouraging results: when Prabakar et al. adapted an ESC protocol for IPC culture and applied it to UCB-MSCs they generated expanded populations of undifferentiated IPCs expressing the key pancreatic TFs PDX1, NGN3, Neuronal Differentiation 1 (NEUROD1), NKX6.1, and Insulin Gene Enhancer Protein ISL-1 “ISL LIM Homeobox 1” (ISL1) [186]. Following transplantation into mice, these cells subsequently differentiated into glucose-responsive IPCs [186]. Zhao et al. took a different approach to exploiting stem cells for T1D treatment, instead focusing on their capacity to downregulate immune responses. The authors achieved reversal of the autoimmune response in NOD mice by transferring autologous Tregs that had been co-cultured with human UCB-MSCs; this led to increased insulin secretion, reduced hyperglycemia and preservation of islet architecture [187–189].

Despite promising signs in rodent studies, the potential of UCB-MSCs treatment for T1D in humans has yet to be fully realized. Haller et al. attempted the first autologous UCB-MSCs transplantation in recently-diagnosed T1D patients in 2008: early indications were encouraging, with transplanted patients showing slowed loss of endogenous insulin production and an increase in peripheral blood Treg cells after 6 months [190]. However, a subsequent study by the same group found no significant difference in C-peptide levels after autologous transfusion of UCB-MSCs combined with oral docosahexaenoic acid and vitamin D supplementation [191]. Similarly, in a non-randomized controlled trial in seven new-onset T1D children who underwent autologous UCB-MSCs infusion, there was no evidence of improvements in metabolic regulation or immune function at the one-year follow-up [192].

The possible reasons for the failure of UCB-MSCs to effectively halt the autoimmune progression in human subjects’ trials, could be the inadequate number of cells with immunomodulation capacity being transferred to T1D patients, or due to the ongoing autoimmune reactions especially in new-onset T1D patients that may comprise memory T-cells, refractive to regulation by Tregs, that enhance the autoimmune destruction of β-cells [193]. Merging transient immune depletion agents with consequent infusion of expanded UCB Tregs may effectively balance the environment of Tregs and effector T cells in T1D patients. Finally, more controlled and randomized clinical trials are crucial to further improve the transplantation process and to investigate the mechanism of UB-MSC survival and behavior in live bodies overtime. Further investigations with larger sample sizes will be important to understand how to translate the successful application of UCB-MSCs infusion from mouse to human.

Cord blood is not the only source of stem cells within the human umbilical cord; Wharton’s jelly is a mucoid connective tissue in the umbilical cord that can also serve as a source of clinically-relevant MSCs (Wharton’s jelly-derived mesenchymal stem cells, WJ-MSCs) for both IPC derivation and immunosuppression [194]. Briefly, WJ-MSCs collection occurs at the time of delivery and avoids the known adverse effects associated with adult stem cell collection from the bone marrow or adipose tissue. Furthermore, features including a high WJ-MSCs proliferation rate, an immune privileged status, minimal associated ethical concerns, and non-tumorigenic capacity render these cells an excellent option to be used in regenerative medicine applications [195].

One of the first studies to use β-cell-like cells derived from WJ-MSCs tested their effects following transplantation into patients with new-onset T1D [196]. Interestingly, a concurrent study suggested that the WJ-MSCs might restore the function of β-cell in T1D patients but it could be affected by the patient’s ketoacidosis history [197], though the underlying mechanism to support this has not yet been tested. A genetically and chemically combined approach for WJ-MSCs induction into IPCs has also been shown to improve the cells’ homing efficiency to the pancreatic gland of diabetic rats [198]; taken together with a growing body of clinical data, these findings may help optimize the use of differentiated WJ-MSCs in T1D.

Undifferentiated WJ-MSCs also have the capacity to induce a protective immune-suppressive state in animal models of T1D and in patients. A study in mice performed by Tsai et al. showed that undifferentiated WJ-MSCs implanted into NOD mice both differentiated into IPCs in vivo, leading to islet repair and maintaining levels of C-peptide and insulin production, and induced beneficial immunosuppression [199]. Such evidence in rodents has since led to the initiation of human trials. A safety and dose-escalation trial is ongoing: in the first stage, Carlsson et al. are carrying out WJ-MSCs allotransplantation into newly-diagnosed (< 2 years) T1D adult men with dose-escalation to establish safety parameters; in the second double-blinded, parallel, placebo-controlled stage, a cohort of T1D patients (men and women) will undergo WJ-MSCs allotransplantation aiming to achieve immunosuppression and preserve endogenous insulin production [200]. Altogether, comparing WJ-MSCs, UCB-MSCs [201] and BM-MSCs [202], it seems that WJ-MSCs are the better anti-diabetic agents, being more homogenous and having greater potential to initiate pancreatic regeneration.

## Medical nutrition therapy in managing T1D

A healthy lifestyle including eating pattern beside pharmacotherapy are major components of managing T1D. For many diabetic patients, determining what to eat is the most challenging part of the treatment plan. Effectual nutrition therapy interventions may be an element of a comprehensive T1D education package or an individualized session [203]. Furthermore, T1D individuals on multiple daily insulin doses, the main focus for nutrition therapy must be on how to adjust insulin doses based on scheduled carbohydrate intake [204, 205]. Reported HbA1_C_ from medical nutrition therapy (MNT) decreases are similar or greater than what would be expected with currently available pharmacologic therapies for T1D [206]. Rigorous insulin management education programs that include MNT have been shown to reduce HbA1_C_ up to 1.9% at 3–6 months, in addition to significant improvement in quality of life over the time [203, 207]. There is no “one-size-fits-all” eating pattern that could work collectively for all T1D individuals, nutritional therapy should be individualized and supervised under the care of a dietitian based on the heath goals, personal favorites and access to healthy options should be considered [208, 209].

## Remaining obstacles and future directions

Marked progress has been made in the past decade towards both personalized diagnosis and treatment for T1D, but significant obstacles and research gaps remain between the current state of knowledge and its translation into widespread clinical benefit. As in many other diseases, the precision medicine for T1D is a new and growing field. Increases ethical, social and legal issues and the necessity to find precise ways to protect subjects’ privacy and confidentiality of their health data. In addition, patients need to know and understand the associated risks and expected benefits of being part of precision medicine research, which requires researchers to create a meticulous approach of obtaining informed consent to recruit participants to research studies. Furthermore, cost-effectiveness of precision medicine approaches comparing to the current standard of care is a gap that needs to be resolved. The impact of diabetes on healthcare systems has been evaluated as the largest contributor to entire healthcare costs. For example, in a study performed by Stedman et al. (reviewed in [34]), the differences between T1D/T2D and non-diabetes subjects in connection to hospital and associated costs in in England. In summary, T1D individuals demanded five times additional secondary care support than non-diabetes subjects. The analysis shows that extra cost of running of hospital services due to their diabetes comorbidities is £3 billion over that for non-diabetes, within this figure, T1D has three times as much cost impact as T2D, suggesting that supporting patients in diabetes management may considerably decrease hospital activity, in addition, the possibility and potential for precision treatment in diabetes is massive, yet profound understanding is missing. It will be vital to decide when and how the application of therapeutics in precision diabetes medicine improves outcomes in a cost-effective style.

Much of our current knowledge of personalized therapeutic approaches to treat T1D comes from experiments in animal models; but a recurring theme in the T1D therapy field is the lack of translation between promising results in mice and the same outcome in humans. Mice are most commonly used for these experiments but exhibit both macroscopic and microscopic differences in pancreatic physiology and T1D pathophysiology. For example, rodents islets have a distinct core structure comprising 60–80% β-cells, 15–20% α- cells, < 10% δ-cells and < 1% PP cells [210–212]; while human islets tend to have ~ 50% β-cells, ~ 40% α- cells, ~ 10% δ-cells and < 5% P-cells [213, 214]. In addition, notable differences in the repertoire of receptors and long non-coding RNAs between mouse and human beta cells have been identified [215]. In terms of modeling T1D, the NOD mouse has long been the approach of choice for majority of pre-clinical and translational invasive studies [216]. The main strength of the NOD mouse is the presence of spontaneous autoimmunity leading to T1D [118, 216] however, in the mice, this is triggered by the insulin antigen, while in humans this phenomenon is more complex, involving several inducing antigens followed by hyperglycemia [217, 218]. Taken together, extreme caution must be exercised when attempting to draw conclusions from animal models and apply them to the human situation [219].

Despite advances in the various therapies discussed above, an ongoing challenge in T1D treatment is the extreme heterogeneity in patients’ disease triggers, prognosis, pathological pathways and thus the response to treatment [220–223]. Important research in human populations has revealed previously unappreciated heterogeneity within the T1D patient population. This has two major implications: firstly, that we are unlikely to discover a “one-size-fits-all” therapy able to cure every case; and secondly that personalized diagnosis is a necessary pre-requisite for personalized treatment. The first step towards this will be the routine assessment of T1D subtype in newly diagnosed patients, including screening for monogenic T1D as well as autoantibody testing to distinguish idiopathic T1D, and, in future, genetic profiling to inform potential gene therapy or stem cell approaches.

In diabetes, the precision medicine approach has been inspired by work including that of Zhao et al., who first developed stem cell educator therapy where T1D patients’ lymphocytes are briefly separated from the blood and co-cultured with UC-MSCs within a closed-loop-system, before being returned to the patient; this treatment dramatically improved metabolic control, reversed autoimmunity and promoted β-cell regeneration [143]. Al-Anazi et al. used a similar approach to try and treat multiple myeloma in 45 adults with T1D who had undergone autologous HSCs; surprisingly the patients were also cured of their diabetes and became insulin-independent [144].

In fact, the next step towards stem-cell-mediated precision medicine for T1D is likely to involve the incorporation of gene therapeutic approaches, synergizing existing stem cell knowledge with advances in cellular and genetic engineering techniques, such as nuclear transfer and genome editing. Moreover, an emerging understanding of the TFs and epigenetic processes that control pancreatic islet lineage-commitment [224], as well as the role of microRNAs in driving cell lineage differentiation [225] are beginning to unlock new knowledge on T1D pathogenesis [226, 227], and are opening fresh possibilities in β-cell generation [228–230].

Together these factors can all be used towards designing a successful protocol for precision medicine in T1D. Alongside, the reframing of T1D as primarily a metabolic disorder (rather than an autoimmune condition) that reflects the combined genomic and environmental landscape of the patient, has facilitated the discovery of new therapeutic targets and diagnostic/prognostic biomarkers [231, 232]. Finally, the ongoing discovery of new and important influences on diabetic pathology, such as the role of gut microbiota [233], and the latest perceptions into the mechanism of T1D and the accumulated recent data that being translated into prospects for tissue-specific prevention trials toward eliminating progressive β-cell loss [234], continues to add to our understanding of this important disease, and thereby our ability to rationally design and test novel interventions with the promise of the future eradication of T1D.

## Data Availability

Not applicable.
